# Deposition of Atmospheric Nitrogen Compounds in Humid Tropical Cuba

**DOI:** 10.1100/tsw.2001.391

**Published:** 2001-12-01

**Authors:** Osvaldo Cuesta-Santos, Arnaldo Collazo, Antonio Wallo, Roberto Labrador, Maria Gonzalez, Paulo Ortiz

**Affiliations:** Atmospheric Environment Research Center, Meteorological Institute, Havana, Cuba

**Keywords:** acid deposition, nitrogen compounds, wet deposition and oxidized nitrogen

## Abstract

Acid deposition, a direct effect of gaseous air pollutants, is causing widespread damage to terrestrial and aquatic ecosystems. Further, these pollutants are responsible for the corrosion of building materials and cultural objects, as well as having an impact on human health. In Cuba, main atmospheric deposition of nitrogen compounds varies from approximately 12.0 to 65.0 kg N ha(-1) year(-1) in rural areas. Ammonia and ammonium are the most important elements in Cuba's tropical conditions.

## INTRODUCTION

Acid deposition remains an important environmental issue in Europe and North America. Furthermore, it is emerging in new geographical areas, including parts of South and Central America, eastern and southern Asia, and southern Africa. In these areas, emissions of nitrogen oxides (NOx) are increasing rapidly as industrialization proceeds and the use of fossil fuels increases[1].

In many areas, soil acidification from nitrification of ammonium deposited from the atmosphere is comparable to that from the deposition of nitric acid[2]. Currently ammonia ($NH_3$) emissions are the same magnitude as NOx and sulphur dioxide ($SO_2$) emissions and are potentially even more acidifying.

Another important link between the acidification and climate change is the additional greenhouse effect caused by increased concentrations of tropospheric ozone, which have occurred during the last decades, especially in the northern hemisphere. This increase is caused by emissions of NOx, carbon monoxide, and volatile organic compounds (VOCs) including methane ($CH_4$). NOx also play an essential role in the total oxidizing efficiency of the atmosphere. The eutrophication effects of nitrogen deposition can increase carbon storage in marine sediments and forest soils, which are likely to be an important component in the global carbon budget.

The nitrogen compounds are NOx (nitrogen dioxide and nitric oxide), ammonia ($NH_3$), nitrate ($NO_3^-$), and ammonium ($NH_4^+$), in aerosols and rainfall. This paper discusses the deposition of atmospheric nitrogen compounds and the trend of their concentrations at several stations along the Island of Cuba between the years 1985 to 2000.

## MATERIALS AND METHODS

To prepare this paper, we utilized data from the Meteorological Institute of Cuban Ministry of Science, Technology, and Environment's network of air pollution control stations. The operation of this network (Fig. 1) is conducted by methodologies recommended by the World Meteorological Organization (WMO) for the monitoring and chemical analysis of the above-mentioned compounds at the regional level. Among the studied stations, three present rural characteristics: La Palma, Colón, and Falla. Two others are subject to urban influence: Casablanca and Santiago de Cuba.

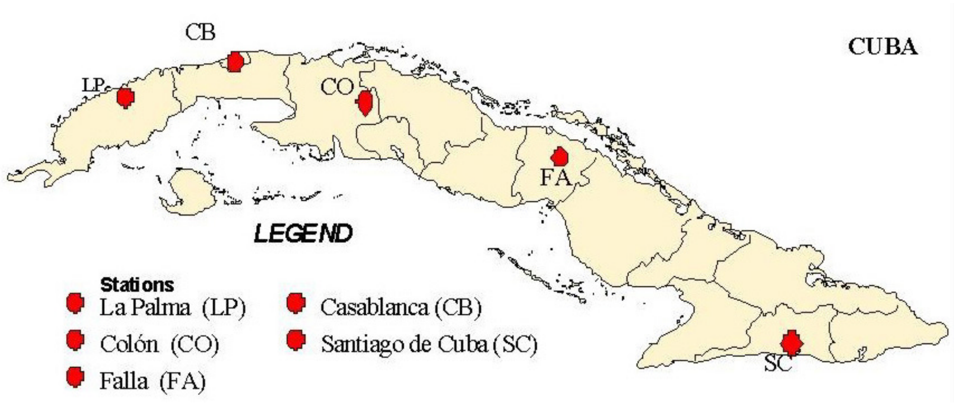

**FIGURE 1.** Nitrogen compound monitoring stations.

In the calculation of wet and dry deposition of atmospheric nitrogen, the concentrations expressed in mg/m$^3$ for $NO_2$, NO, $NH_3$, $NO_3^-$, and $NH_4^+$ were converted into nitrogen (N). Concentrations in rainfall are expressed in mg/l.

Samples were monitored and analysed using WMO-approved methods for rainfall, aerosol, and gases. All methods were quantitatively determined colorimetrically. Nitrates react in the acidified sample with 2.4 xylenol, which is then extracted with synthetic toluene and finally re-extracted from the synthetic toluene with sodium hydroxide solution. Ammonium ion concentration is reacted with hypochlorous acid, phenol, and a manganous sulphate solution. The blue indophenol formed is quantitatively determined colorimetrically. Atmospheric $NH_3$ was collected by passing it through dilute sulphuric acid, and determined by minor modification of the indophenol technique[3].

Because of known deficiencies in existing monitoring methods for dry deposition flows, their recalculation through the deposition velocity[4,5,6,7] and the obtained concentrations from monitoring was undertaken. The mean heavy concentrations of wet deposition flows for each year (starting from summary monthly samples) and the rainfall quantities were used for both concentrations. Deposition flows are expressed in kg-N/ha$^{-1}$ year$^{-1}$. For the selection of the values of deposition velocity, data reported by different authors were analysed and those adapted to the climatic conditions of Cuba were chosen.

Two statistical techniques were used to analyze the concentration trend behavior. One of them is aimed at the trend's detection and another at its modeling. Both techniques are based on the Mann-Kendall test, which allows determination of the trend, as well as retrogressive analysis to define at what moment the change of the trend began[7]. The Mann-Kendall test consists of the progressive determination of the test's statistics[8]. In addition, the atmospheric N compounds were selected, statistics of which gave a significant trend. For this trend, the equation was obtained through simple regression analysis. All methods were performed in SPSS Plus statistical system.

Another aspect studied was the trend of the concentrations during these years. This allows the behavior of both natural and anthropogenic sources of the above-mentioned elements to be known.

## RESULTS AND DISCUSSION

The total deposition values (dry and wet) for the main nitrogen compounds at several of the studied stations are reflected in Fig. 2. The Casablanca station presents the highest deposition values for $NH_3$ and $NO_2$ due to the nearness of potent sources of air pollution in the industrial zone of Havana City. The Colón station presents the highest values of ammonium in the rainfall. The intensity of sources of N compounds and the large rainfall at this station are due to its geographical location in the middle of our country and its use as a farming and livestock zone. Farming is

**TABLE 1**
**Values Selected of the Deposition Velocity in cm s$^{-1}$ for the Gases and Aerosols in Dependency of Land Use and Climatic Conditions**

| Station | $NO_2$ | NO | $NH_3$ | $NO_3^-$ | $NH_4^+$ |
|---|---|---|---|---|---|
| La Palma | 0.5 | 0.25 | 1.5 | 0.5 | 0.5 |
| Colon | 0.5 | 0.25 | 1.5 | 0.2 | 0.2 |
| Falla | 0.5 | 0.25 | 1.5 | 0.2 | 0.2 |
| Casablanca | 0.8 | 0.1 | 1.0 | 0.1 | 0.1 |
| Santiago de Cuba | 0.8 | 0.1 | 1.0 | 0.1 | 0.1 |

recognized as a major source of atmospheric $NH_3$ in Europe and contributes about half of the global $NH_3$ emissions[9]. The $NH_4^+$ value is also high at the Casablanca station, but its main source is Havana City.

In general, these annual values are similar to those reported in most of Europe and North America[10,11,12], with the exception of the large urban and industrialized centers, where the deposition in Europe is significantly higher. The annual values at studied stations vary from a minimum of 9.9 kg-N/ha$^{-1}$ year$^{-1}$ recorded in La Palma station in 1996, which has significantly low values of $NH_3$, to a maximum of 35.5 kg-N/ha$^{-1}$ year$^{-1}$ in 1995 at the Casablanca station, where the $NH_3$ deposition was considerably higher. As can be appreciated in our tropical conditions, the natural and anthropogenic emissions of $NH_3$ are very important in the biogeochemical cycle of nitrogen.

On a worldwide level, approximately 50% of the nitrogen species emitted to the atmosphere are deposited on the Earth's surface by rainfall[13]. For instance, the relative importance of dry and wet deposition of nitrogen and sulphur compounds depends on many factors, such as climate and surface features.

In Cuba, as indicated by data collected at the studied stations, wet deposition (Fig. 3) represents approximately 50% of the total nitrogen deposition, though as a general rule the dry deposition is greater, with the exception of the Colón station, where the wet deposition represents 55%. The Casablanca station is more greatly influenced by the urban area, and dry deposition represents 60% of the total. For our region, therefore, the wet deposition is considered significant and also depends on characteristics of our tropical climate.

The greatest air-sea fluxes of oxidized nitrogen occurs in the North Atlantic Ocean, Europe, and Africa[14], while the reduced nitrogen forms, mainly $NH_4^+$ and $NH_3$, make a contribution of about 40% of total N flow into oceans from the atmosphere[15].

The values (Fig. 4) attained for Cuba that refer to the main forms of oxidized nitrogen show that they represents on average 23% of the total N deposition. The stations with higher anthropogenic influence (Casablanca and Santiago de Cuba) receive greater amounts of oxidized nitrogen compounds. The Falla station currently receives the largest proportion of acid deposits. On the other hand, the stations at Colón and La Palma receive the lowest amount of oxidized nitrogen due to the majority of its contributions from natural sources of reduced nitrogen.

These values reflect a very large percentage of the $NH_3$ deposition, so it is possible that the ammonia neutralizes atmospheric acidity in the short term, thereby preventing damage due to acid deposition; on the other hand, ammonia can cause soil and groundwater acidification over moderate to longer periods of time. Due to urban influence, the station that receives the greatest contribution of nitrates via rainfall is Casablanca.

The La Palma station is representative of the forest ecosystem of Cuba's western region and update receives approximately 20% from acid deposits. Because of its highly acidic soil (pH 4.1–4.5), this region will be very sensitive to acid deposition. This can produce harmful ecological risks in soil productivity, affecting the growth of trees in forested areas as well as the productivity of some others crops. Forest areas one able to receive from five to six times more dry deposition (gas and aerosol) by the greater deposition velocity than these generate[3], all above makes this region ecologically more sensitive. The answer and reaction of forest ecosystem to ambient chemical changes are complex.

On the other hand, some nitrogen compound concentrations showed a significant trend in the period from 1985 to 2000. As can be seen for $NO_2$ values, the monitoring stations with highest anthropogenic influence, such as Casablanca and Santiago de

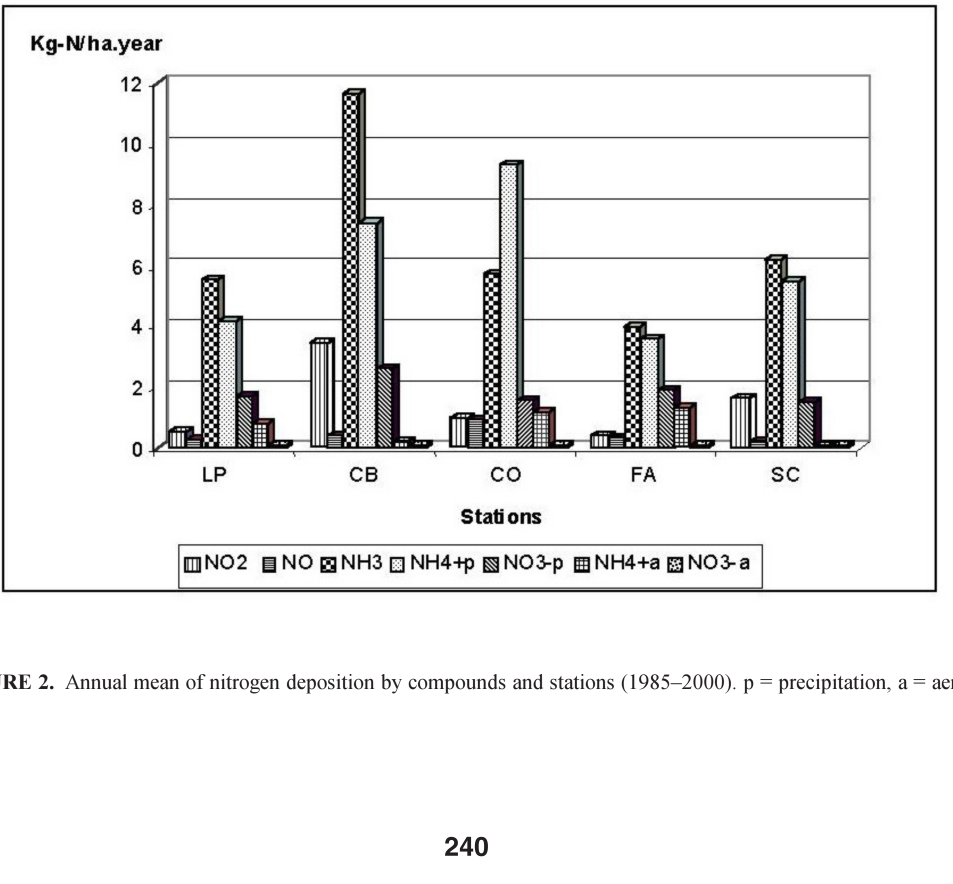

**FIGURE 2.** Annual mean of nitrogen deposition by compounds and stations (1985–2000). p = precipitation, a = aerosol

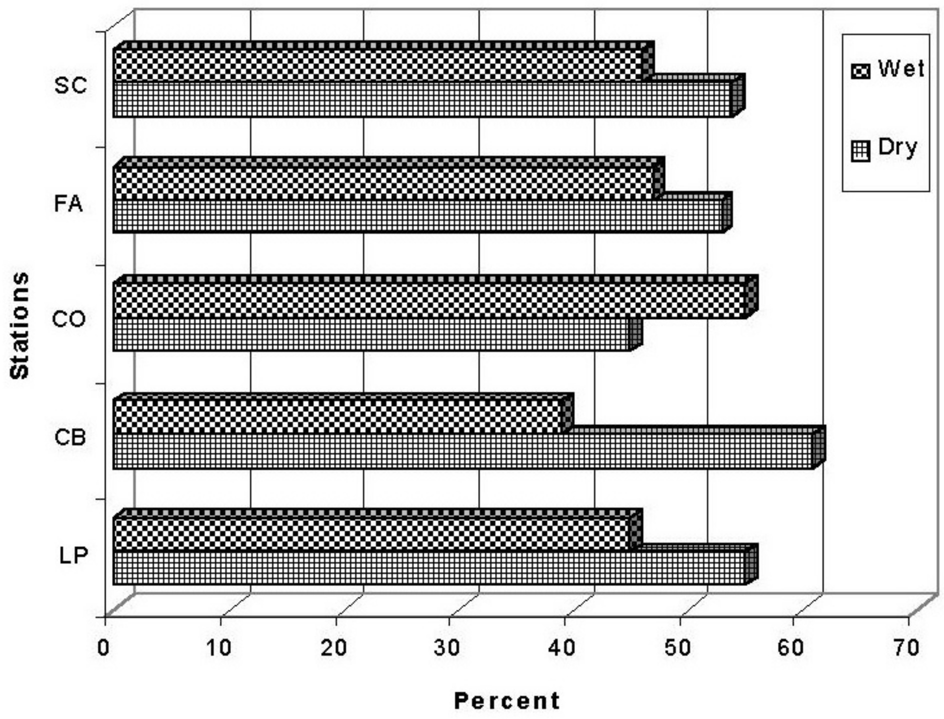

**FIGURE 3.** Relationships between wet and dry deposition.

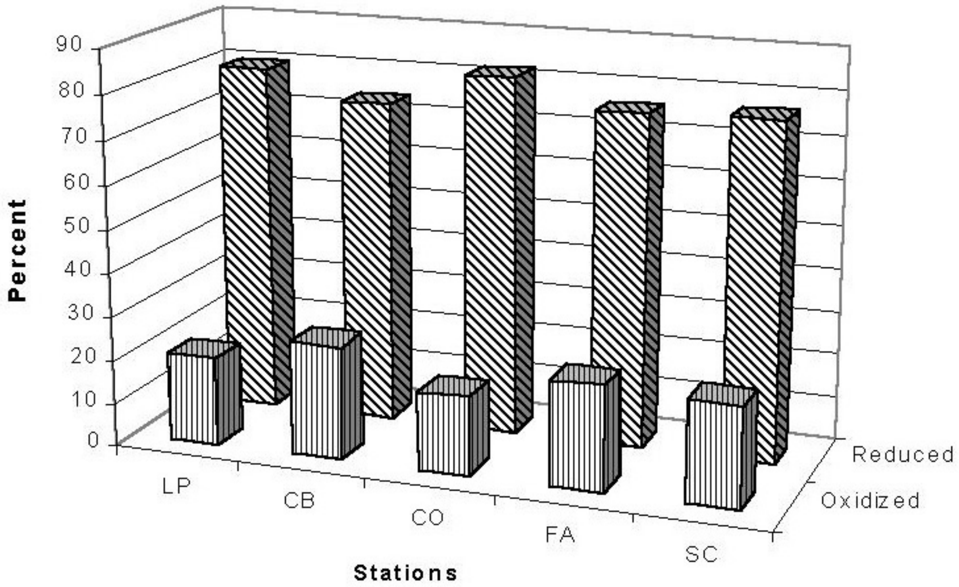

**FIGURE 4.** Relationships between oxidized and reduced forms.

Cuba, have experienced a trend toward the decrease of concentrations because of the slowing of industrial production and transportation in Cuba since 1989 (Fig. 5).

This influence also seems to affect the La Palma station, which receives the highest oxidized compound deposition among rural stations (Figs. 6 and 7). On the other hand, the Colón rural station presents a significant trend toward an increase in $NO_2$ and $NH_3$ concentrations, and the Falla station also shows increases (Fig. 8). These two rural stations seem to be influenced by biomass being burned for energy purposes. It is also possible that

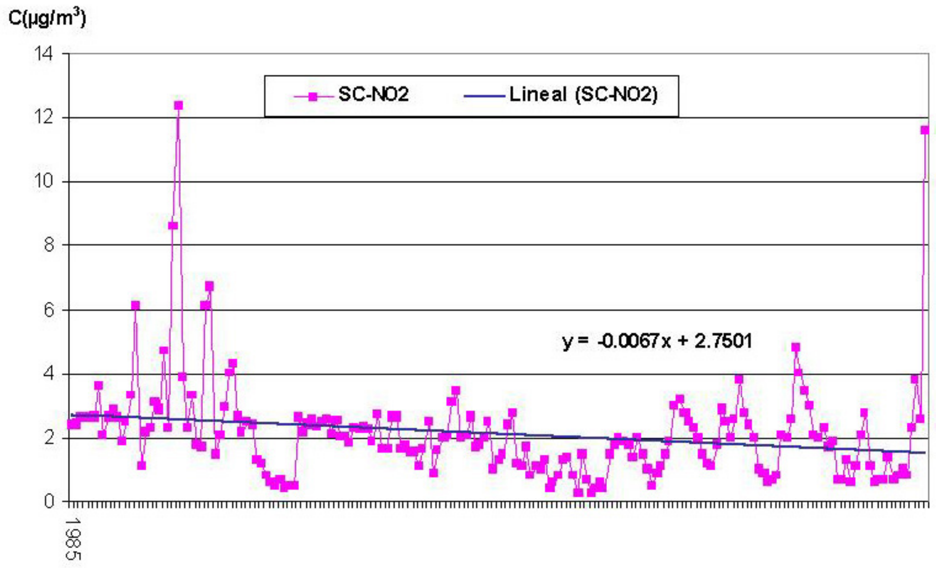

**FIGURE 5.** The $NO_2$ concentration trend at the Santiago de Cuba (SC) station.

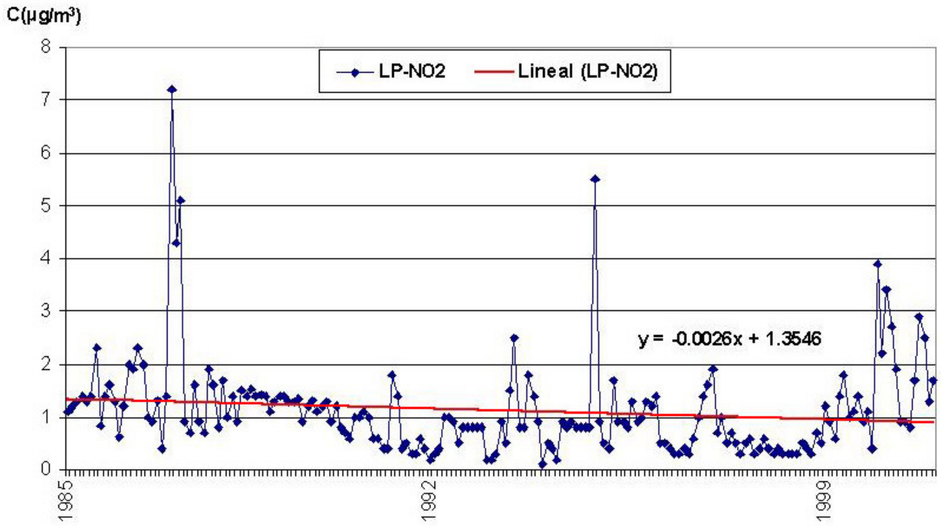

**FIGURE 6.** The $NO_2$ concentration trend at the La Palma (LP) station.

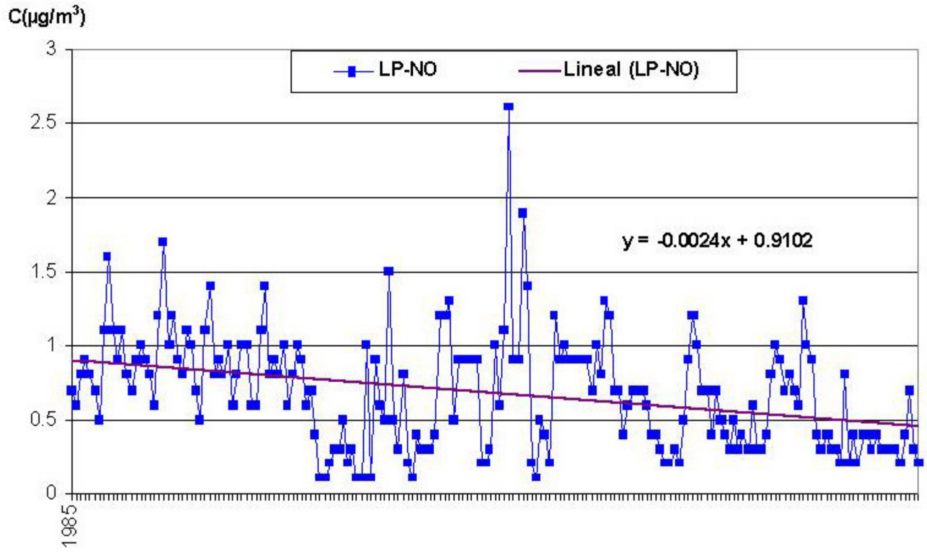

**FIGURE 7.** The NO concentration trend at the La Palma (LP) station.

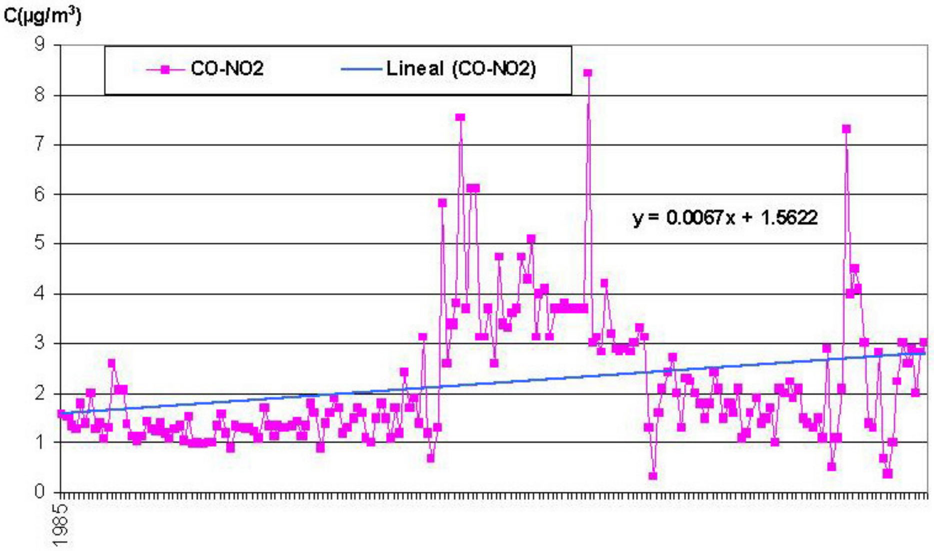

**FIGURE 8.** The $NO_2$ concentration trend at the Colón (CO) station.

changes in land use and application of fertilizers have influenced this increase. In any case, the tendency toward an increase of ammonium aerosols is of interest in regard to all studied stations and this increase is particularly significant in the rural stations of La Palma and Colón, mainly as a result of the use of ammonia fertilisers in these regions.

Nitrate particles exhibit an increasing trend at the La Palma and Casablanca stations. This has possibly been caused by long-range transport[16] of substances that serve as $NO_x$ reservoirs. In addition, the increasing trends in ammonium aerosol amounts is of interest in all studied sites, because it reaches a significant trend to increase in the La Palma and Colón rural stations, mainly due to use of ammonia as fertilizer in these areas.

In relation to rainfall, nitrate levels exhibit the same behavior as $NO_2$ at these stations. However, only the Casablanca station demonstrates a significant trend toward increase in nitrate concentrations, while ammonium in rainfall shows an increasing trend only at the La Palma and Falla stations, both of which are considered rural.

Damage caused to materials exposed to the atmosphere constitutes one of the most important direct effects of acidifying air pollutants[17]. One of the potentially more pronounced effects of deposition in stations with anthropoid influence is the accelerated increase of atmospheric corrosion, which results in the deterioration of limestone and sandstone, materials used in buildings, sculptures, and monuments of great cultural value. Acid deposition also causes the deterioration of stained-glass windows, papers, and cultural objects as well as some types of plastics and metallic materials used indoors.

According to oxidized species deposition of the Casablanca and Santiago de Cuba stations, these areas will suffer greater impact due to acidification of the soil, on account of the areas' urban activity of the soil, fact that is given to urban activity. However, the Santiago de Cuba region could be much more affected, because its soils are highly acidic (pH 4.6–5.0) and therefore more sensitive to acid deposition.

On the other hand, the Colón and Falla stations present a trend toward increasing oxidized nitrogen compounds[7]; if this continues, it may affect crops sensitive to acidity, such as beans and tomatoes. At the same time sugar cane is also sensitive to acid deposition, and the zones in question are highly exploited for the cultivation of this crop, which may gradually suffer a loss of yields as the elements are deposited.

Another potential effect that can cause deposition of oxidized nitrogen compounds is acidification of freshwater surface streams and lakes[1]. This results in a reduction of bicarbonate ions and an increase of nitrate concentrations, which produces an increase of concentrations of certain metals in these water sources. Metals such as aluminium, cadmium, zinc, lead, and mercury are very toxic and can be ingested by different forms of aquatic life through alimentary chains, and may eventually make their way up the food chain to humans.

Studies of effects produced by acid deposition must be conducted in an harmonic and integral way between different specialists concerned with the protection of the environment and natural resources; this is the only way to preserve the ecological balance and biodiversity that our planet requires.

## CONCLUSIONS

In Cuba, the total deposition for main nitrogen compounds varies from 9.9 to 35.5 kg-N/ha$^{-1}$ year$^{-1}$. Dry deposition of N represented here is somewhat higher than 50%. $NH_3$ has a great weight in dry deposition. Weights of both depositions depend on characteristics of our rainy, tropical climate. Meanwhile, reduced forms of nitrogen contribute approximately 80% of the total deposition; that which is linked with the characteristics of our climate and the diverse natural sources for these compounds.

In stations subject to urban influence, the behaviour of $NO_2$ and nitrate in rainfall is similar. In the rural stations of La Palma, Colón, and Falla, it is necessary to maintain a systematic monitoring, because a trend toward increasing concentrations of oxidized nitrogen compounds could in the future cause potential damage to agriculture and forests in these regions.

Harmful potential effects due to acid deposition impact our aquatic and terrestrial ecosystems, through corrosion of materials and in human health; because of this, a system of integrated monitoring must be imposed to enable us to recognize and mitigate its effects.

## ACKNOWLEDGEMENTS

We would like to thank the Cuban Environmental Agency for financial support that enabled the accomplishment of this work.

| | | |
|---|---|---|
| Received: | July | 10, 2001 |
| Revised: | November | 8, 2001 |
| Accepted: | November | 13, 2001 |
| Published: | December | 1, 2001 |
